# Editorial: Probiotics in Children Health

**DOI:** 10.3389/fped.2022.918877

**Published:** 2022-06-07

**Authors:** Baldassarre Maria Elisabetta, Laforgia Nicola, Campeotto Florence, Salvatore Silvia

**Affiliations:** ^1^Department of Biomedical Science and Human Oncology, “Aldo Moro” University of Bari, Bari, Italy; ^2^Department of Interdisciplinary Medicine, “Aldo Moro” University of Bari, Bari, Italy; ^3^Pediatric Gastroenterology Department, APHP Necker-Enfants Malades Hospital, Paris, France; ^4^Department of Pediatrics, Ospedale “F. Del Ponte”, University of Insubria, Varese, Italy

**Keywords:** probiotics, children, health, neurocognitive abilities, preterm/full term infants

There is a growing body of evidence linking human health to the activity and composition of gut microbiota as well as its alteration to intestinal and systemic diseases. In the last years, functional gastrointestinal disorders, inflammatory, allergic, and autoimmune conditions, metabolic syndrome, and neurological and behavioral diseases have all been associated with microbiota unbalance, the so-called “dysbiosis” (1). The perinatal period and early life should be considered as extremely vulnerable ages for the establishment of physiological microbiota and its consequent programming of immune and metabolic responses.

Full term neonates suffering from mild asphyxia show, during the first day of life, a significant predominance of *Lachnospiraceae* and *Clostridia*, in which both species are correlated with poor neural development and communication scores at 6 months of life (Zhang et al.). This negative effect is due to harmful microbial by-products with impaired intestinal permeability and activation of the inflammatory cascade which cause gut-brain axis damage after the hypoxic-ischemic insult. In preterm neonates, various factors, including cesarean section, drugs, prolonged hospitalization, parenteral nutrition, and reduced breast feeding favor a dysregulated microbiota. Moreover, preterm infants are frequently colonized by nosocomial antibiotic-resistant bacteria with a significant risk of infections and a negative impact on neonatal growth and development (2). To favor the development of “good” microbiota, several strategies have been explored and the use of probiotics has dramatically increased, although the optimal strain still needs to be identified. It is noteworthy that the supplementation of *Bifidobacterium longum* subsp. *infantis* (*B. infantis*) EVC001 to preterm neonates has recently been shown to increase both intestinal *Bifidobacteria* and the functional capacity to utilize human milk oligosaccharides and to reduce enteric inflammation and the development of antibiotic resistance (Nguyen et al.). In children admitted to pediatric intensive care units, sepsis has been associated with proliferation of opportunistic pathogenic bacteria and with a reduction of commensal flora, *Bifidobacteria*, short chain fatty acids, and microbial diversity. Moreover, the presence of *Enterococcaceae* correlates with positive blood inflammation biomarkers (Liu et al.). Whether dysbiosis is either the cause or the consequence of severe infections needs to be clarified in order to evaluate the role of gut microbiota as a prognostic biomarker and to develop targeted intervention. In a randomized trial, bovine lactoferrin alone or in combination with *Lactobacillus rhamnosus* GG (LGG) reduced the incidence of late-onset sepsis in very low birth weight neonates (3). In a prospective, randomized, double-blind, placebo-controlled study, that enrolled 156 children, bovine lactoferrin was instead ineffective in preventing antibiotic associated diarrhea (Wronowski et al.).

The disruption of gut microbiota and different profiles of short chain fatty acids produced by unhealthy diet and lifestyle have been linked to the development of obesity and metabolic complications, such as liver steatosis and insulin resistance. As dysbiosis may produce excessive energy storage and adiposity and altered gut peptides, hormones, and proinflammatory status, manipulation of microbiota with prebiotics and probiotics may promote energy homoeostasis and regulate appetite (through glucagon-like peptide-1, peptide YY, and modified ghrelin secretion) and glucose sensitivity (Petraroli et al.).

A recent study examined 160 healthy children from birth to 12 years and investigated the relationship between gut microbiota with body mass index and found that microbial richness with relative abundance of *Firmicutes* and *Bacteroidetes* of genera *Alistipes* and *Subdoligranulum* producing propionate and butyrate was negatively correlated with BMI during childhood (4).

Microbiota can be influenced by various environmental and dietary factors and it is also considered as a therapeutic target for allergies (5). LGG has been given for atopy prevention and to promote tolerance to cow's milk protein in allergic children (6). LGG mainly acts through regulation of intestinal and systemic immunity and restores intestinal permeability. A systematic review and meta-analysis including 10 randomized controlled studies by Tan et al. showed the efficacy and safety of LGG in children under 3 years of age with cow's milk allergy compared to controls [risk ratio (RR), 2.22; 95% confidence interval (CI), 1.86–2.66; moderate-quality evidence]. LGG reduced fecal occult blood (risk ratio, 0.36; 95% CI, 0.14–0.92; *p* = 0.03; low-quality evidence), while no significant difference of the dermatitis score was found. LGG and other probiotic strains have also been proposed for management of pain in infants and children with functional gastrointestinal disorders (7). Despite the absence of underlying anatomic and biochemical abnormalities, these common gastrointestinal manifestations causing parental concern, high use of both drugs and healthcare resources, and impaired physical and psychological wellbeing have been associated with dysbiosis, Germ-free animal models, fecal transplantation, and human studies have demonstrated that microbiota influences the cross-talk of the gut-brain axis and affects the response to inflammation, stress, and pain stimuli with bad microbiota linked to hyperalgesia, chronic mood, and neurological disturbance (8, 9). Shaping the microbiota and the production of short chain fatty acids with a targeted nutritional intervention and selected probiotics, since early life, may modulate the cytokine profile, intestinal and blood-brain permeability, metabolism and level of many neurotransmitters and neuroactive molecules (such as tryptophan, serotonin, GABA, dopamine), vagal circuits, microglial function, brain development, synaptogenesis, and cerebral plasticity (Larroya et al.).

Nevertheless, there are no definitive data to recommend the use of probiotics for the prevention of functional gastrointestinal disorder and only a few randomized controlled trials have reported limited efficacy of probiotics in reducing pain (Capozza et al.). Only *L. reuteri* DSM179389 showed a significant reduction of crying and/or fussing duration in cohorts of breast-fed infants, but it is still unclear which are the determinants of this beneficial effect in responsive subjects. Generally, the heterogeneity of the studies, in terms of inclusion criteria and enrolled patients, probiotic strain, duration of treatment, and outcome measures, still limit the comparison and interpretation of the results (10).

The recognition of the importance of a balanced microbiota has inspired and will promote research on selected microorganisms and probiotic strains for different health problems, including infections, allergy, obesity, mood, and behavioral and gastrointestinal disorders.

A better understanding of the complex relationship between diet and microorganisms and host immune, metabolic, endocrine, and nervous systems is of pivotal importance to design effective prevention strategies, personalized nutrition, and tailored treatment for communicable and non-communicable diseases in neonates, children, and adults.

## Author Contributions

BE and SS prepared the first draft of manuscript. LN and CF revised the manuscript. All authors contributed to the article and approved the submitted version.

## Conflict of Interest

The authors declare that the research was conducted in the absence of any commercial or financial relationships that could be construed as a potential conflict of interest.

## Publisher's Note

All claims expressed in this article are solely those of the authors and do not necessarily represent those of their affiliated organizations, or those of the publisher, the editors and the reviewers. Any product that may be evaluated in this article, or claim that may be made by its manufacturer, is not guaranteed or endorsed by the publisher.
